# Holiday Heart Syndrome: A Literature Review

**DOI:** 10.7759/cureus.79816

**Published:** 2025-02-28

**Authors:** Juan Daniel Alvarado, Paola Zuniga, Immy Stringer, Allison Ramirez, Erick Cortes, Jhiamluka Solano

**Affiliations:** 1 General Practice, Universidad Católica de Honduras, Tegucigalpa, HND; 2 General Practice, Universidad Nacional Autónoma de Honduras, Tegucigalpa, HND; 3 General Internal Medicine, Scunthorpe General Hospital, Scunthorpe, GBR; 4 Internal Medicine, Hospital General San Francisco, Olancho, HND; 5 Cardiology, Scunthorpe General Hospital, Scunthorpe, GBR; 6 Education Committee, Academy of Medical Educators, Cardiff, GBR; 7 Resident Doctor Committee, Royal College of Physicians, London, GBR

**Keywords:** alcohol-induced arrhythmias, atrial fibrillation, binge drinking, cardiac electrophysiology, holiday heart syndrome

## Abstract

Holiday Heart Syndrome (HHS) is a condition characterised by the development of atrial fibrillation (AF) and other tachyarrhythmias following episodes of binge drinking in individuals without pre-existing cardiac disease. As the binge drinking rate rises worldwide, it is increasingly important to understand the pathophysiology, epidemiology, and clinical significance of this syndrome. The objective of this literature review is to synthesise current evidence on the relationship between binge alcohol drinking and AF, exploring the underlying mechanisms and risk factors associated with HHS. A literature review was conducted using PubMed and Cochrane databases up to August 2024. Articles were selected based on predefined inclusion criteria, focusing on studies assessing the impact of acute alcohol intake on AF incidence. Studies evaluating chronic alcohol consumption, literature reviews, case series, and publications in languages other than English were excluded. A total of 11 studies met the inclusion criteria, comprising cohort and case-control studies. The findings consistently demonstrated a strong association between binge drinking and AF onset. Epidemiological evidence suggests that an increased incidence of new AF cases in individuals without structural heart disease can be attributed to alcohol consumption. Mechanistic insights identify several pathophysiological processes that contribute to the development of HHS, including autonomic dysregulation, ion channel modifications, and transient atrial structural changes. Acute alcohol consumption leads to increased sympathetic activity and reduced vagal tone, increasing heart rate variability and predisposing individuals to AF. Furthermore, alcohol has been shown to increase the activity of T-type calcium channels, which contributes to atrial ectopy and electrical instability. Structural alterations, such as reduced left atrial emptying fraction, have also been observed in binge drinkers, further supporting the link between alcohol and arrhythmogenesis. The evidence reviewed underscores the significant arrhythmogenic risk of binge drinking. While some studies suggest a J-shaped relationship between alcohol intake and AF risk, binge drinking consistently appears as a major trigger. However, variability in study populations and methodologies necessitates further research to establish safe consumption thresholds and interventions. Most studies relied on self-reported alcohol intake, with inconsistent screening methods, and physiological assessments included electrocardiogram monitoring and blood alcohol level measurements. This review highlights the significant role of binge drinking in the pathogenesis of HHS, stressing the need for targeted public health interventions and personalised patient counselling. Future research should prioritise longitudinal studies to improve risk assessment models and clarify the long-term cardiovascular impacts of alcohol-induced AF. Clinicians are encouraged to routinely screen for alcohol use, particularly in patients with a history of arrhythmias, to help prevent recurrent episodes and minimise associated complications.

## Introduction and background

Holiday Heart Syndrome (HHS) refers to the onset of tachyarrhythmias following episodes of binge drinking, defined as the consumption of five or more standard drinks within two hours. The World Health Organization (WHO) indicates that the global prevalence of binge drinking stands at 18.2% [1]. Alcohol consumption is prevalent in Western nations, with approximately 53% of Americans consuming alcohol regularly and 44% of these individuals engaging in binge drinking [2]. Observational studies suggest that new-onset atrial fibrillation (AF) cases in individuals without structural heart disease may be associated with alcohol consumption, particularly binge drinking [3], even among those without pre-existing cardiovascular disease. These findings suggest that alcohol, particularly ethanol, has a direct arrhythmogenic effect [4,5].

Patients with HHS typically present with palpitations characterised by a rapid and irregular heartbeat. Other symptoms may include chest pain, shortness of breath, dizziness, and syncope [6]. The diagnosis of HHS primarily relies on clinical history and the temporal relationship between alcohol consumption and symptom onset. Electrocardiography (ECG) is essential to document the arrhythmia, typically revealing AF or other supraventricular tachyarrhythmias [7]. The cornerstone of HHS management is the cessation of alcohol consumption. In many cases, arrhythmias resolve spontaneously within 24 hours after abstaining from alcohol. For persistent arrhythmias, medical interventions, such as rate or rhythm control, may be necessary, and anticoagulation therapy should be considered based on individual risk factors for thromboembolism [8].

Proposed mechanisms by which acute alcohol intoxication may predispose to tachyarrhythmias include shortening of the atrial action potential and refractory period, conduction slowing, oxidative stress, mitochondrial dysfunction, apoptosis, and autonomic disturbances. Excessive alcohol consumption may also lead to myocardial injury and inflammation [5]. Despite the well-documented association between alcohol and tachyarrhythmias, the precise mechanisms remain poorly understood [9]. Existing research remains limited, leaving significant gaps in our knowledge of how alcohol exacerbates atrial arrhythmias. To address this, we conducted a literature review to synthesise current findings and provide a clearer, updated understanding of this prevalent yet under-researched condition. 

## Review

Methodology

We conducted a literature review in PubMed and Cochrane, published until August 2024, using 'holiday heart syndrome' as the primary search term. We obtained 78 articles and conducted an independent peer review and selection of the articles. Initially, articles were selected based on title and abstract, followed by conflict resolution by the independent reviewers. In total, three independent reviewers conducted the screening process. In cases of disagreement, a consensus-based approach was used, and, if conflicts remained unresolved, a senior reviewer adjudicated decisions based on predefined inclusion and exclusion criteria.

**Figure 1:**
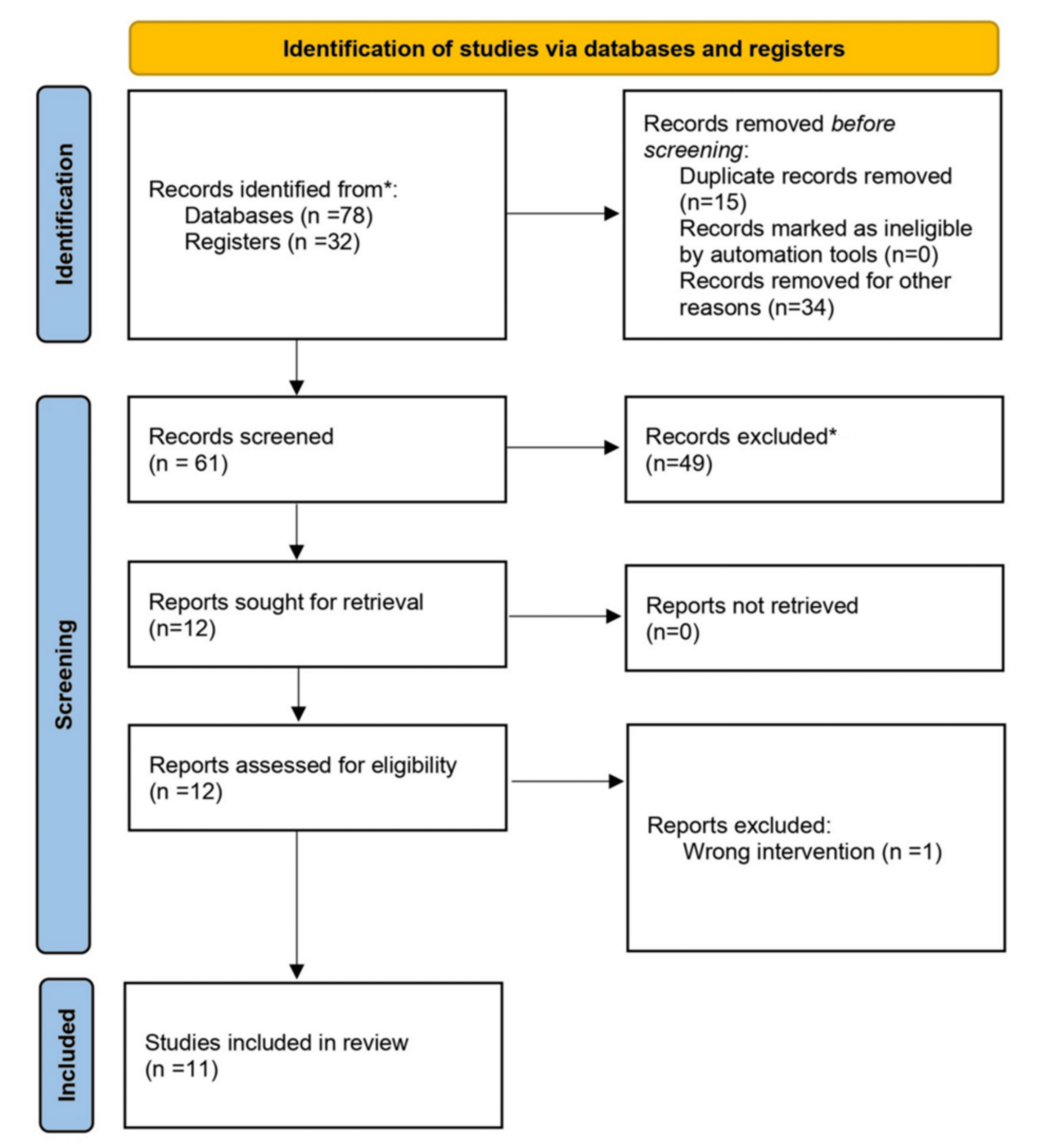
PRISMA flowchart *PubMed and Cochrane

Articles were selected for further inclusion after full-text appraisal. We included additional studies found in the references of the identified articles. Citation tracking was systematic, following a snowballing method to identify relevant studies from reference lists. The additional articles were appraised using the same method described. After independently analysing the articles, we identified 11 relevant articles concerning HHS and acute alcohol intake. We included RCT, non-RCT, cohort studies, and case-control studies conducted in adult patients with HHS and no known cardiovascular disease. We excluded literature reviews, systematic reviews, case reports, editorials, experimental studies, and case series. Additionally, we excluded articles not published in English and those focusing on chronic alcohol intake (Figure 1).

As this was a clinical review, we did not systematically include experimental animal or cellular studies for mechanistic insights. We acknowledge this as a limitation, as such studies could provide additional context on the underlying pathophysiology of alcohol-induced arrhythmias.

Results

A total of 11 studies met the inclusion criteria, including cohort and observational studies, all examining the relationship between acute alcohol consumption and new onset of AF in individuals without known cardiac conditions (Table 1) [10-20]. While experimental studies were excluded from the primary analysis, certain studies, like Brunner et al. [19], were referenced to provide contextual mechanistic insights. Across these studies, binge drinking was consistently associated with an increased risk of AF episodes. Smaller observational studies demonstrated significant acute autonomic changes following alcohol consumption, whereas larger epidemiological datasets reinforced the statistical association between alcohol intake and AF risk [12,17,18]. Additionally, studies such as Brunner et al. [19] used heart rate variability (HRV) analysis to demonstrate autonomic dysfunction, while Voskoboinik et al. [17] observed increased heart rate and reduced HRV within 24 hours of binge drinking.

**Table 1:** Characteristics of the appraised studies

Study	Year	Country	Study Design	Sample Size	Key Findings	Limitations
Larsson et al. [10]	2014	USA	Prospective Cohort	79,019	High alcohol intake (>15 drinks/week) increases atrial fibrillation (AF) risk. Binge drinking (>5 drinks/occasion) raises the risk by 29%.	Lacks mechanistic data
Tu et al. [11]	2022	USA	Retrospective Cohort	403,821	J-shaped relationship: lowest AF risk at 5 drinks/week. Heavy drinking increases AF risk, especially in women consuming spirits.	Self-reported alcohol intake, potential recall bias
Ettinger et al. [12]	1976	USA	Retrospective Cohort	20	AF episodes linked to whiskey and beer consumption. ECG changes persisted in some patients.	Small sample size, lacks generalisability
Engel and Luck [13]	1983	USA	Prospective Cohort	14	Whiskey promotes AF; higher blood alcohol levels favour atrial fibrillation over flutter.	Very small sample size
Liang et al. [14]	2012	Canada	Cohort	30,433	Regular alcohol intake increases AF risk, even without binge drinking. Binge drinking significantly raises AF risk.	Observational study, no causal inference
Rich et al. [15]	1985	USA	Retrospective Cohort	1,396	Heavy drinkers have a higher AF incidence. Alcohol withdrawal contributes to arrhythmias.	Potential selection bias
Cohen et al. [16]	1988	USA	Case-Control	3,966	Drinking >6 drinks/day doubles the risk of supraventricular arrhythmias.	Self-reported intake, possible misclassification bias
Voskoboinik et al. [17]	2022	Australia	Prospective Cohort	50	Binge drinking (8.3 ± 3.1 drinks) triggers AF episodes within 24 hours, increases heart rate, and decreases heart rate variability.	Small sample, short follow-up period
Kupari and Koskinen [18]	1991	Finland	Retrospective Cohort	289	AF is more common after weekend alcohol consumption - strong correlation with excessive alcohol intake in the prior 48 hours.	Small sample size, retrospective design
Brunner et al. [19]	2021	Germany	Experimental	15	Increased sympathetic activation and heart rate post-alcohol. Prolonged QT interval and altered autonomic balance.	Experimental design, limited clinical applicability
Brunner et al. [20]	2017	Germany	Observational	3,028	Arrhythmias occurred in 30.5% of acute alcohol intake cases. Chronic alcohol use had no significant AF effect.	Potential residual confounding

The combined sample size across these studies ranged from small observational cohorts of 15 participants to large-scale epidemiological studies with over 400,000 individuals. This variability in study populations allowed for a broad assessment of the effects of alcohol on cardiac rhythm, capturing both short-term physiological responses and long-term trends in AF incidence.

The studies identified suggest multiple underlying mechanisms for HHS. Several studies highlighted the effect of heightened sympathetic activity and reduced vagal tone following alcohol binge drinking, leading to increased HRV and atrial ectopy. Others reported direct electrophysiological effects, which may predispose individuals to AF. The relationship between alcohol dose and AF risk was also examined, with some studies indicating a J-shaped association. This suggests that moderate alcohol consumption may not carry the same risk as binge drinking. However, all studies concurred that excessive alcohol intake, particularly within a short period, was a significant trigger for AF.

History of HHS

HHS was first described in the 1970s, following Ettinger's observation that individuals consuming large volumes of alcohol (often in the context of festival celebrations, hence the condition's name) were presenting with acute-onset tachyarrhythmias. This was despite a significant lack of any structural or metabolic cardiac history [4]. The seminal case series reviewed the admission of 24 individuals (20 men and 4 women). They described the recurring association of new dysrhythmias following a short period of excessive alcohol intake, with resulting cardiac conduction delays and left ventricular impairment identified months later. Normal cardiac chamber pressures and angiography, however, were found following treatment, suggestive of an early cardiac response to alcohol-induced damage. This was a new concept, given the previous opinion that arrhythmias occurred secondary to electrolyte, endocrine, structural, or valvular impairment and myopathy secondary to chronic rather than acute alcohol consumption.

In this study, from which the term 'HHS' was first coined, AF was the most common arrhythmia identified. Still, atrial flutter, atrial tachycardias, junctional tachycardias, and ventricular tachycardias were also observed [4]. Notably, a key aspect of the definition revolved around the cessation of symptoms and ECG findings with abstinence from alcohol. All 24 individuals in the study experienced HHS-related arrhythmias, clarifying that there were no control subjects included in the analysis. This series followed work Ettinger's group had completed with animal models previously. In 1971, it had taken up to 10 weeks of ethanol infusion to see minor histological changes in myofilaments in mice myocardium [21], whilst no abnormalities in atrial conduction were noted in dogs subjected to acute ethanol infusions in 1976 [12]. These findings were initially thought to contradict the idea of alcohol-induced arrhythmias, as no immediate conduction abnormalities were observed in experimental models. However, Ettinger's 1978 paper, which primarily focused on humans with a history of repeated alcohol use, provided clinical evidence that acute alcohol ingestion could indeed stimulate new atrial arrhythmias, particularly in individuals without pre-existing cardiac disease.

Other papers had previously described the changes found in the heart following chronic alcoholism [21]. A 1959 Lancet paper described isolated attacks of dyspnoea and palpitation as 'common in alcoholic myocardial disease,' with episodes of AF recognised at a time when it was 'mostly obscure and relatively rare' if cardiomyopathy was not ischaemic in origin [22]. A body of literature detailed the cardiac pathologies that chronic alcoholism can cause, with multiple reviews since warning of the risk of continued alcohol consumption to cardiac health, e.g., dilated cardiomyopathy [8,23]. Studies published at a similar time to the first detailing HHS found evidence that the onset of cardiac arrhythmias was related to the duration and amount of alcohol consumed prior in the context of binge drinking [16,24-26]. Epidemiological studies up to the early 2000s, nicely summarised by Balbão et al. [26], loosely concluded that chronic alcohol consumption increased AF risk, whilst meta-analyses have often shown an association with AF if daily alcohol intake is high, as opposed to binge drinking specifically [27,28]. It is worth noting that most studies primarily examine chronic alcohol consumption, with binge drinking typically analysed as a subgroup.

Studies conducted beyond the period of initial recognition, however, have since demonstrated similar evidence of new-onset arrhythmias in people binge drinking during these holidays, alongside those who regularly indulged in excess alcohol [13,15,28-30]. In many cases, these involved young individuals <60 years of age, in whom the majority spontaneously reverted to sinus rhythm, or athletes with no other precursor for new electrical conduction deficits [15,29,31]. In the majority of cases, anticoagulation was not prescribed, and further episodes of arrhythmia were not identified.

The management of such cases and the consideration of the long-term risk posed to the heart have been inconsistent in the reported studies. Given the incidence and evolving interest in AF in particular, attributed to the different foci described triggering AF [26], theories behind conduction mechanisms (described later), or therapeutics and interventional strategies available to treat it, HHS continues to be a relevant topic of study. A recent review published in 2022 by Linz et al. [32] highlighted the difficulty there has been in examining isolated cases of HHS and how an overwhelming body of literature assessing the effects of chronic alcohol use overshadows the impact of binge drinking and the progression of our understanding of it in recent years.

Discussion

Our study examined the link between binge alcohol consumption and AF, commonly referred to as HHS. The relationship between alcohol intake and arrhythmias, particularly AF, is complex and multifaceted. Research indicates that both acute and chronic alcohol consumption can significantly influence cardiac rhythm. This review consolidates findings from key studies on alcohol-related AF risk, offering insights into underlying mechanisms, epidemiological patterns, and clinical implications. While the evidence strongly associates binge drinking with AF onset, variations in risk patterns across different populations highlight the need for further investigation.

Epidemiological Trends and Conflicting Risk Associations

Several large-scale cohort studies support a direct relationship between alcohol intake and AF. Liang et al. [14] found a linear increase in AF risk proportional to alcohol consumption, with risk increased for binge drinking and chronic alcohol use. However, Tu et al. [11] identified a J-shaped relationship, suggesting a lower AF risk with moderate intake (five to seven drinks per week) compared to abstinence, but a sharp increase with any further excessive consumption. These variations may stem from study design differences, population characteristics, and potential confounders, such as overall cardiovascular health and genetic predisposition.

Kupari and Koskinen [18] emphasised the acute effects of binge drinking, with AF incidence rising significantly following weekend alcohol consumption (>150 g/48 hours). This was especially true in individuals with a prior history of AF, who experienced episodes of AF 11-34 hours after binge drinking. Rich et al. [15] found that heavy alcohol use was more prevalent among AF patients than controls, and alcohol withdrawal contributed to arrhythmia onset. In contrast, the findings from Brunner et al. [19,20] reported that chronic alcohol consumption did not show a significant association with arrhythmias other than sinus tachycardia. This discrepancy highlights the need for further investigation into the long-term electrophysiological effects of alcohol, considering differences in study design, population characteristics, and confounding variables.

Cohen et al. [16] found that consuming more than six drinks per day doubled the risk of supraventricular arrhythmias. Similarly, Voskoboinik et al. [17] linked binge drinking (8.3 ± 3.1 drinks per occasion) with sympathetic activation and reduced HRV, predisposing individuals to AF. Ettinger et al. [4] highlighted that AF episodes were predominantly observed in whiskey and beer drinkers, with ECG changes persisting in some cases.

The link between binge drinking and an increased risk of electrical abnormalities, such as AF, is well established. However, our review revealed a growing interest in whether even low to moderate alcohol consumption could also contribute to these abnormal cardiac events [10]. While some evidence suggests the possibility of a 'safe' threshold for alcohol consumption with respect to AF, further research is needed to confirm its existence. Many of the studies reviewed have excluded populations with significant comorbidities; this affects the generalisability of findings by limiting the applicability of results to broader, real-world populations. Many individuals who consume alcohol, particularly heavy drinkers, often have underlying cardiovascular conditions, such as hypertension, diabetes, or structural heart disease, which independently contribute to AF risk. By focusing on healthier individuals without pre-existing conditions, studies may underestimate the true burden of alcohol-related AF in clinical practice. In reality, alcohol consumption may act synergistically with these comorbidities to further increase AF risk, making the observed associations in controlled study populations less reflective of real-world scenarios. This exclusion may lead to an underestimation of alcohol’s arrhythmogenic potential, particularly in vulnerable individuals who may experience compounded effects due to their underlying health conditions. 

The inconsistency in study findings may stem from differences in study design, the populations studied, or geographical variations. Given these uncertainties, recommendations on alcohol consumption should be individualised, especially for patients at risk of AF. In contrast, patients at equal risk of AF who drink little or no alcohol may die before the AF reveals itself [33]. The paradoxical nature of these findings demonstrates the challenge of drawing a clear line on the health impacts of alcohol, particularly for those who are at risk for AF. Low consumption of red and white wine and very low consumption of spirits may not be associated with an increased risk of AF; in contrast, any intake of beer or cider may be associated with harm [11]. A high intake of any alcoholic beverage was consistently associated with greater AF risk across the studies analysed. Still, these observations suggest that perhaps even a social dose of ethanol can be harmful to those at risk for AF or flutter, as in the presence of sinus node or valvular disease [13]. This reinforces the idea that the threshold for safe alcohol consumption is not universal and should be tailored to individual health profiles.

Pathophysiological Mechanisms Underlying Alcohol-Induced AF

The pathophysiology of HHS is multifactorial, involving autonomic dysfunction, ion channel dysregulation, and transient structural changes in the heart. Several studies have elucidated the mechanisms by which acute alcohol consumption predisposes individuals to AF, highlighting the complex interplay between autonomic nervous system imbalances, ion channel modifications, and mechanical alterations in atrial function.

Autonomic dysfunction plays a critical role in the arrhythmogenesis of HHS. Acute alcohol exposure disrupts the autonomic nervous system by inhibiting vagal tone while simultaneously stimulating the sympathetic nervous system, leading to excessive catecholamine release from the adrenal medulla and nerve endings [8]. This autonomic imbalance results in increased heart rate, reduced HRV, and heightened atrial ectopic activity, all of which are known to predispose individuals to AF. Two studies here demonstrated significant decreases in deceleration capacity (a marker of parasympathetic activity) and changes to heart rate and QT interval, related to increases in blood ethanol concentration [17,19]. Engel and Luck [13] similarly described shortened atrial refractory periods in patients exhibiting disorganised atrial activity following whiskey ingestion. One of the key metabolites of alcohol, acetaldehyde, further exacerbates this imbalance by selectively inhibiting cardiac vagal tone and augmenting sympathetic activity, creating an arrhythmogenic substrate for AF onset.

Beyond autonomic dysfunction, binge drinking influences atrial excitability through ion channel modifications. Excessive alcohol consumption has been shown to upregulate T-type calcium channels (TCCs) via the NFAT/PKC/GSK3β signalling cascade, as demonstrated by Wang et al. [33]. This increased expression of TCC isoforms Cav3.1 and Cav3.2 enhances atrial ectopy, further elevating AF susceptibility. Additionally, transient structural changes contribute to the pathophysiology of HHS. No significant alterations in atrial volume or ventricular function have been noted, and echocardiography following AF episodes was unremarkable in subjects exhibiting 'HHS,' according to Rich et al. [15]. Voskoboinik et al. [17] reported that binge drinking leads to atrial mechanical dysfunction, specifically a reduction in left atrial emptying fraction. These temporary structural impairments reinforce the link between excessive alcohol consumption and arrhythmogenesis, emphasising the intricate relationship between autonomic nervous system alterations, ion channel regulation, and transient cardiac structural changes in the development of AF.

Clinical Considerations and Preventative Strategies

Given the well-established connection between binge drinking and AF, clinicians should routinely assess alcohol consumption in patients, particularly those with a history of arrhythmias. A proactive approach to AF prevention involves multiple strategies, beginning with public health initiatives that raise awareness of alcohol-induced arrhythmias and promote safer drinking behaviours. Educational campaigns, emphasising the cardiovascular risks associated with excessive alcohol intake, could play a crucial role in reducing AF incidence.

Further considerations should be given to anticoagulation strategies in patients with recurrent alcohol-related AF. While standard CHA_2_DS_2_-VASc score-based recommendations remain the cornerstone of stroke prevention, additional risk factors related to alcohol-induced variability in AF episodes should be taken into account. Individualised risk assessments should be implemented to identify high-risk individuals and develop tailored prevention strategies. Clinicians should encourage patients with prior AF episodes or predisposing factors to practice moderation or abstain from alcohol altogether. By integrating these targeted interventions into routine clinical practice, the burden of alcohol-induced AF may be significantly reduced.

Gender and Age Variability in Alcohol-Related AF

Regarding sex and age, the identified studies did not find a significant interaction between alcohol consumption and gender, as directionally consistent associations were observed in both men and women. It is important to note that the majority of participants included across all studies were male, and the proportion of men was often higher in groups deemed heavy drinkers for analysis. However, Tu et al. [11] found that women consuming spirits had a notably higher AF risk than those consuming wine, indicating potential metabolic and hormonal influences on alcohol-induced arrhythmogenesis. Additionally, many studies focused on younger, healthier individuals (<60 years), potentially underestimating the effects of alcohol on older adults with cardiovascular comorbidities.

Study limitations

A key limitation of this literature review is the inability to perform a meta-analysis, primarily due to heterogeneity in methodologies, sample sizes, and outcome measures among the included studies. The variation in how alcohol consumption was assessed, combined with differences in participant populations and study designs, makes it challenging to derive pooled estimates or establish clear dose-response relationships. This restricts the generalisability of findings and highlights the need for more robust and standardised research approaches.

Confounding factors further limit the interpretation of findings in this review. Many included studies did not fully account for potential confounders, such as concurrent medication use, electrolyte imbalances, genetic predisposition, or underlying cardiovascular conditions - all of which could influence AF risk independently of alcohol intake. Additionally, lifestyle factors such as smoking, caffeine intake, and stress levels were inconsistently reported, making it challenging to isolate alcohol as the sole contributor to observed arrhythmias. The presence of these confounders may have led to an overestimation or underestimation of the true effect of alcohol consumption on AF risk.

Furthermore, reliance on self-reported alcohol intake introduces potential biases, including underreporting or recall inaccuracies. The lack of standardised screening tools, such as AUDIT-C or CAGE, further limits the comparability of findings across studies. Although some studies incorporated objective physiological measurements, such as ECG changes and HRV, these assessments were not consistently applied, limiting their utility in forming broader conclusions.

## Conclusions

Our review highlights the significant role of binge drinking in the development of AF and the risk it poses to healthy individuals engaging in binge drinking, as well as those drinking low or moderate amounts of alcohol on a regular basis. Future research should prioritise large-scale prospective cohort studies that track alcohol consumption patterns and AF incidence over time, incorporating validated screening tools such as AUDIT-C and CAGE to improve consistency in data collection. Additionally, studies focusing on high-risk populations, including individuals with hypertension, diabetes, or structural heart disease, are necessary to better understand the synergistic effects of alcohol and comorbidities on AF risk. Finally, mechanistic and translational studies exploring the cellular and molecular effects of alcohol on atrial tissue would provide critical insights into the underlying pathophysiology of alcohol-induced AF.
